# Synthetic Poly(lactic-co-glycolic Acid) Microvesicles as a Feasible Carbon Monoxide-Releasing Platform for Cancer Treatment

**DOI:** 10.3390/membranes11110818

**Published:** 2021-10-26

**Authors:** Wen-Jyun Wang, Chung-Dann Kan, Chih-Yen Chen, Yi-Yao Meng, Jieh-Neng Wang, Wei-Ling Chen, Chia-Hsiang Chen, Wei-Peng Li

**Affiliations:** 1 Department of Medicinal and Applied Chemistry, Kaohsiung Medical University, Kaohsiung 807, Taiwan; courtesy0120@gmail.com (W.-J.W.); a0970641257@gmail.com (Y.-Y.M.); 2 Division of Cardiovascular Surgery, Department of Surgery National Cheng Kung University Hospital, College of Medicine, National Cheng Kung University, Tainan 704, Taiwan; kcd5086@gmail.com; 3 Department of Materials and Optoelectronic Science, National Sun Yat-Sen University, Kaohsiung 804, Taiwan; cychen@mail.nsysu.edu.tw; 4 Department of Pediatrics, National Cheng Kung University Hospital, College of Medicine, National Cheng Kung University, Tainan 704, Taiwan; jiehneng@gmail.com; 5 School of Biomedical Engineering, College of Biomedical Engineering, Taipei Medical University, Taipei 110, Taiwan; lynnchen.k@gmail.com; 6 Institute Food and Drug Administration, Ministry of Health Welfare, Taipei 115, Taiwan; 7 Department of Medical Research, Kaohsiung Medical University Hospital, Kaohsiung 807, Taiwan; 8 Drug Development and Value Creation Research Center, Kaohsiung Medical University, Kaohsiung 807, Taiwan

**Keywords:** microvesicles, poly(lactic-co-glycolic acid), carbon monoxide, emulsion, cancer

## Abstract

Biogenic microvesicles (MVs) play a pivotal role in intercellular signal communication, thus initiating critical biological responses such as the proliferation of cancer cells, gene and protein transport, and chemo-drug resistance. In addition, they have been recognized as having great potential in drug delivery applications. However, the productivity of biologically produced MVs is not sufficient for clinical applications. In this study, synthetic poly(lactic-co-glycolic acid) (PLGA) MVs were prepared via a double emulsion method. The PLGA MVs had a biogenic MV-mimic vesicular structure with a hydrophilic core/surface and hydrophobic interior of the shell, showing great potential for drug delivery. We successfully embedded hydrophobic iron carbonyl (IC), a carbon monoxide (CO) donor, in the PLGA shell region, enabling the delivery of IC in an aqueous solution. Because of the intrinsic properties of PLGA, it was susceptible to temperature, and the MVs could easily collapse in a warm environment, leading to the decomposition of IC into CO. The in vitro result indicated that the cell viability of A549 lung carcinoma cells significantly decreased to 14% after treatment with IC-loaded PLGA MVs for 24 h, suggesting that these synthetic PLGA MVs constitute an excellent drug delivery platform.

## 1. Introduction

Extracellular vesicles (EVs) are biological nanoparticles secreted by mammalian cells, bacteria, and fungus under specific conditions or environments [1,2,3]. Microvesicles (MVs) and outer membrane vesicles (OMVs) are two kinds of EVs that have definite characteristics—namely, a phospholipid bilayer membrane-constructed vesicular sphere with a specific size range (100–1000 nm) and equipping of the original membrane proteins, which are generated through outward budding from animal cells and bacteria, respectively [4,5]. The main objective of secreting these EVs is to communicate between individual cells through the signaling genes or proteins delivery, thus regulating cellular processes such as tumor metastasis, gene transport, defense, infection, quorum sensing, and autophagy [6,7,8,9]. Based on this principle, MVs and OMVs have been indicated to have great potential as carriers to transport drugs into specific cancer cells; therefore, many scientists have performed related research on identifying vesicle types, exploring the mechanism of EV secretion, and developing the methodology of vesicle generation [10,11,12]. The generation of MVs and OMVs by biogenesis has shown very low efficiency, and the amount of product produced has been insufficient so far for clinical application. Therefore, developing a feasible method with the ideal productivity of vesicles remains a great challenge that it is essential to overcome.

As an alternative approach, artificially preparing an EV-mimic carrier is an ideal way to directly overcome the low yield of vesicles from the biogenic method [13,14]. Many biocompatible polymers, fatty acids, and lipids are excellent materials for preparing vesicles [15,16]. A drawback of synthetic EV-mimic carriers is that they lack natural biological cargo and thus cannot provide any additional biofunctions that real EVs could offer. On the other hand, vesicular carriers with a membranous structure have shown great affinity with cells and an excellent capability to load a high quantity of drugs, which makes them a suitable drug delivery system (DDS) [17].

Carbon monoxide (CO) is a silent killer that is colorless, odorless, and capable of causing severe harm [18]. Inhaling excess CO can cause hypoxic poisoning and even shock, as CO has a more than 250 times greater affinity for hemoglobin than oxygen and thus inhibits O_2_ delivery. On the other hand, CO has also shown a unique ability to upregulate cancer cells’ metabolism by continually activating mitochondria, thus inducing excess reactive oxygen species (ROS) generation and leading to the exhaustion of mitochondria and the apoptosis of cancer cells, which is called the anti-Warburg effect [19]. Inorganic complexes composed of metal centers and multicarbonyl ligands that can release large amounts of CO gas, usually termed as carbon monoxide-releasing molecules (CORMs), provide safer means of CO delivery than the direct inhalation of CO gas [20]. However, CORMs usually present high toxicity, unideal stability in the bioenvironment, and poor water solubility. Therefore, developing a suitable DDS for stabilizing and delivering CORMs into the target area after administration has remained a challenge and necessity.

Here, we used an easily handled double emulsion method to obtain synthetic poly(lactic-co-glycolic acid) (PLGA) MVs with a clear vesicular structure. The shell was constructed by an amphiphilic copolymer via a water-in-oil-in-water (W/O/W) emulsion process. The resulting MVs had a lipid bilayer-mimic membrane feature showing a hydrophilic membrane surface and a hydrophobic shell interior wherein water-insoluble drugs could be embedded. In this study, PLGA MVs were used to load iron carbonyl (IC) as a CO donor, and CO therapy, through providing IC-loaded PLGA MVs to cancer cells, was evaluated to determine its feasibility. Through CO measurement, the vast amount of CO released from the IC-loaded PLGA MVs was detected under warm conditions (37 °C), which can easily be achieved in any tumor site. Our in vitro results indicated significant cytotoxicity to A549 lung cancer cells after incubation with IC-loaded PLGA MVs, which indicated that these synthetic PLGA MVs are an excellent candidate for replacing biogenic MVs as a reliable DDS for the applications of water-insoluble drug delivery and gas therapy.

## 2. Materials and Methods

### 2.1. Materials

All reagents were of analytical grade and used without further purification. Iron carbonyl (IC, Fe(CO)_5_), hemoglobin (MW = 64,500), sodium hydrosulfite (Na_2_S_2_O_4_, 85%), poly(vinyl alcohol) (PVA, MW = 47,000, 98%), 3-(4,5-dimethylthiazol-2-yl)-2,5-diphenylte-trazolium bromide (MTT, C_18_H_16_BrN_5_S, 97.5%), and poly(lactic-co-glycolic acid) (PLGA, 50:50, MW = 24,000–38,000) were purchased from Sigma-Aldrich. Dichloromethane (CH_2_Cl_2_, 99.9%) was bought from ECHO. Water was obtained using a Millipore direct-Q deionized water system throughout all studies.

### 2.2. Preparation of IC-Loaded PLGA MVs

First, an oil phase solution containing 4 mL dichloromethane and 40 mg PLGA was prepared. For the first emulsion, 0.8 mL PVA(aq) (1.7 mM) was slowly added in oil phase solution under sonication at 4 °C for 2 h. During the process of emulsion, micelles w/o PLGA were produced. Afterwards, 100 or 20 μL iron carbonyl solution was mixed with the first emulsion solution. This mixture solution was dropped into another 12 mL PVA(aq) solution for the second emulsion process, which used a homogenizer at 4 °C for 20 min to generate w/o/w IC-loaded PLGA microvesicles. The resulting solution was centrifuged at 1000 rpm for 1 min at least two times to remove unembedded iron carbonyl and residual organic solvent, which stayed at the bottom of the tube. Subsequently, the supernatant was concentrated by centrifugation (1500 rpm, 3 min). The pellet of IC-loaded PLGA MVs was dispersed in ultrapurified water and stored in the refrigerator at 4 °C.

### 2.3. Characterization

The morphology of the MVs was observed by a transmission electron microscope (TEM, Hitachi H-7500) and scanning electron microscope (SEM, Zeiss Auriga). UV−vis spectra were recorded on a UV−vis absorption spectrometer (Analytik Jena SPECORD 200 PLUS). The FT-IR spectra of the MVs were measured by a Fourier transform infrared spectrometer (FT-IR, Bruker ALPHA). The quantification of cell viability was performed using an enzyme-linked immunosorbent assay reader (ELISA reader, BioTek Synergy HTX). A dynamic light scattering spectrometer (DLS, Otsuka Electronics ELSZ-2000) was used to measure the hydrodynamic diameters of MVs.

### 2.4. Evaluation of CO Release from IC-Loaded PLGA MVs

Reduced hemoglobin (r-Hb) was used as an indicator to determine the amount of CO released by observing the absorption peak at 410 nm, which corresponds to the formation of r-Hb-CO. Three milligrams of sodium hydrosulfite were added to 6 mL hemoglobin solution (14.5 μM) in a sealed bottle, then the solution was purged with N_2_ gas for 20 min to obtain an r-Hb work solution. Afterwards, 0.8 mL r-Hb work solution was mixed with 0.2 mL IC-loaded PLGA MV solution (13 mg/mL) and immersed in a warm water bath (37 °C) for different time periods (0, 10, 30, and 60 min) to decompose the IC into CO gases, which could further form r-Hb-CO in the r-Hb solution. The supernatants of r-Hb-CO were obtained by centrifugation (1500 rpm, 3 min), and their absorbances were measured by a UV−vis spectrometer.

### 2.5. Cell Culture

A549 cells (human lung carcinoma/alveolar cell lines) were cultured in DMEM containing 0.1 mM NEAA, 1% penicillin/streptomycin (PS), and 10% FBS in an incubator at 37 °C, 95% humidity, and 5% CO_2_.

### 2.6. In Vitro Cytotoxicity Study

The A549 cells were subcultured in a 96-well plate (4000 cells per well) and incubated for 24 h. The old medium was removed, then fresh medium, without and with PLGA MVs, IC-loaded PLGA MVs, or IC100-loaded PLGA MVs, was added to the culture. UV–vis measurement determined the concentration of materials with an optical density of 0.14 at 800 nm. The cells treated with MVs were incubated for another 24 h, followed by a wash with phosphate-buffered saline (PBS) at least three times. Finally, MTT reagent was used for the determination of cell viability by the standard ELISA method. All data were obtained in quadruplicate.

### 2.7. In Vitro Reactive Oxygen Species Evaluation

APF dye as an indicator was used to evaluate the intracellular ROS level in this study. A549 cells were subcultured in a 96-well plate (4000 cells/per well) and incubated for 24 h. The old medium was removed, and then a fresh medium with APF (10 μM) was added to the culture. After 30 min, this medium with excess APF dye was changed for a new medium without and with PLGA MVs, IC-loaded PLGA MVs, or IC100-loaded PLGA MVs. Materials were concentrated at an optical density of 0.14 at 800 nm, as measured by UV. The cells were incubated for 30 min, and the correlated emission signals were detected by the fluorescence plate reader (Ex = 485 nm; Em = 525 nm). All data were obtained in quadruplicate.

## 3. Results and Discussion

### 3.1. Synthetic Polymer Microvesicles with Cell Membrane-Mimic Shells for Drug Loading

**Figure 1:**
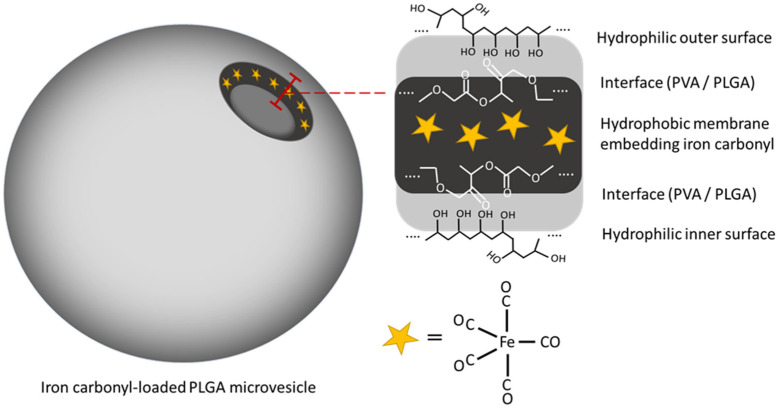
The vesicular structure and membrane-mimicking feature of iron carbonyl (IC)-loaded PLGA MVs. Water-insoluble IC could be embedded in the hydrophobic interior of the shell.

For some simple conditions, such as applying vesicles for drug delivery only, artificial synthetic polymer MVs without the functions of biological reaction and conjugation are ideal for replacing scarce biogenic EVs because they can be easily produced on a gram scale. For this reason, we applied a mature double emulsion approach to prepare PLGA MVs with a similar structural feature to the cell membrane. Amphiphilic PLGA with PVA constructed a biological membrane-mimic shell showing hydrophilic inner and outer surfaces of the shell and a hydrophobic interior. Here, a hydrophobic coordination compound, iron carbonyl, was used as a CO donor and successfully embedded into the interspace of the PLGA shell during the emulsion process. These IC-loaded PLGA MVs could spontaneously release CO in warm surroundings, thus showing great potential for use as a new vesicular carrier for biomedical applications (Figure 1).

### 3.2. Morphology and Size Distribution of MVs with and without IC Loading

**Figure 2:**
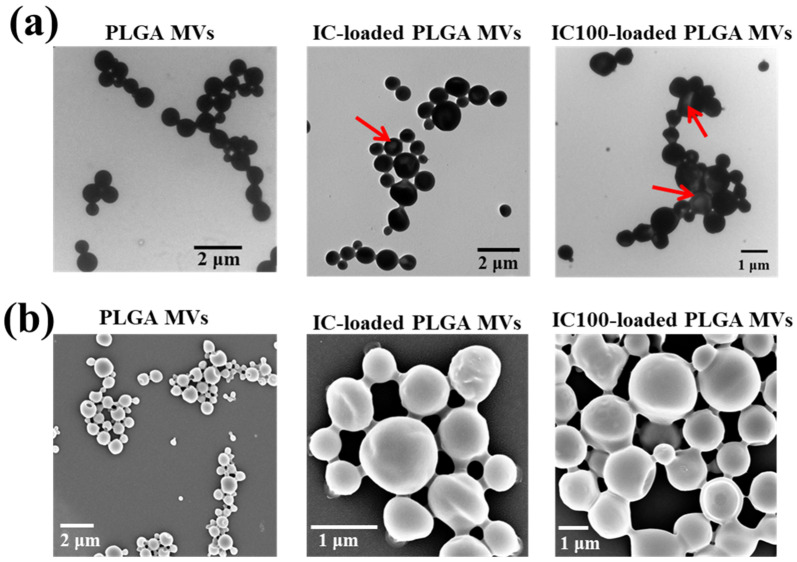
Morphology observation of vesicles. (**a**) TEM images of PLGA MVs, IC-loaded PLGA MVs, and IC100-loaded PLGA MVs. The red arrows indicate regions of brighter contrast. (**b**) HR-SEM images of PLGA MVs, IC-loaded PLGA MVs, and IC100-loaded PLGA MVs.

**Figure 3:**
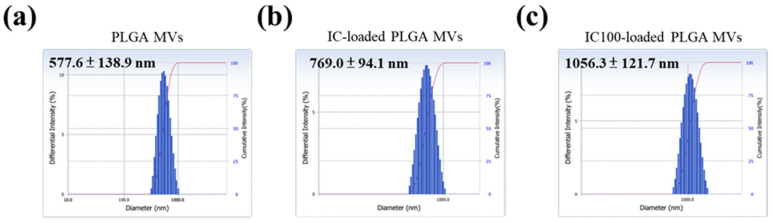
The hydrodynamic diameters of (**a**) PLGA MVs, (**b**) IC-loaded PLGA MVs, and (**c**) IC100-loaded PLGA MVs, as measured by DLS.

In this study, low (20 µL) and high (100 µL) amounts of iron carbonyl were loaded onto PLGA MVs, which were named IC-loaded and IC100-loaded PLGA MVs, respectively. Through observation by transmission electron microscopy (TEM), the PLGA MVs and IC-loaded PLGA MVs showed an obvious spherical morphology and homogenous size distribution (Figure 2a). The TEM image of IC-loaded PLGA MVs revealed a few vesicles with a bright contrast, which indicates inflation of the vesicular shells (Figure 2a). The slight CO gas generation from the high loading of unstable IC after preparation might cause this result. Scanning electron microscopy (SEM) images indicated that all IC-carrying MVs maintained intact vesicular surfaces without collapsing or bursting, even though a small amount of the loaded IC decomposed into gas and increased the stress on the vesicles (Figure 2b). Through DLS measurement, the IC-loaded PLGA MVs showed a centralized particle size distribution of 769.0 ± 94.1 nm. They presented a slightly larger size than the PLGA MVs, which indicates the swelling of the vesicles due to gas generation inside of the carriers (Figure 3a,b). A more severe swelling phenomenon was found in IC100-loaded PLGA MVs, the size distribution of which was 1056.3 ± 121.7 nm (Figure 3c).

### 3.3. Characterization of IC Loading on the MVs

**Figure 4:**
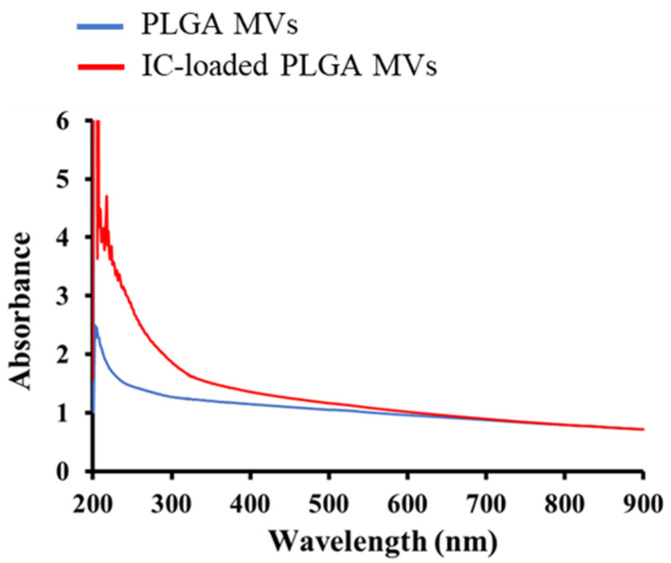
Optical characterization of PLGA MVs and IC-loaded PLGA MVs.

**Figure 5:**
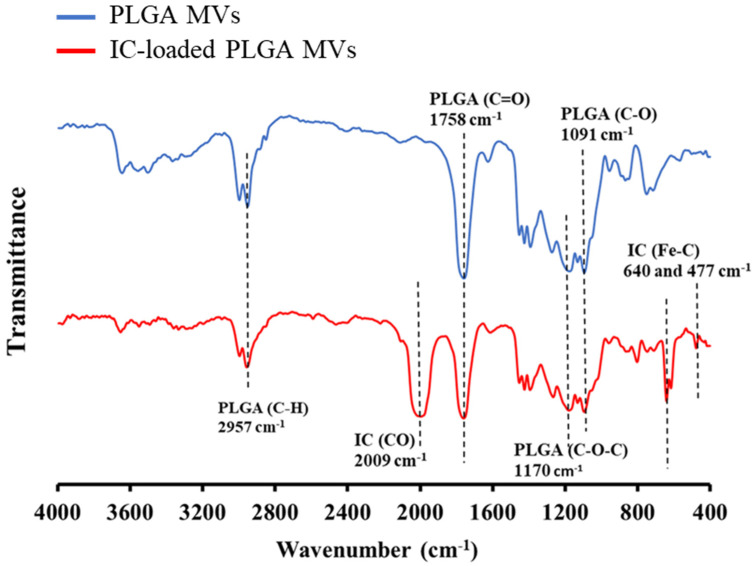
FT-IR analysis of PLGA MVs and IC-loaded PLGA MVs.

The yellow colloidal solution of IC-loaded PLGA MVs indicated that the IC molecules were loaded onto the PLGA carriers, as empty PLGA microparticles showed a white color. UV–vis analysis also indicated that the IC-loaded PLGA MVs showed a higher characteristic absorbance at the wavelength range of 200–500 nm compared to PLGA MVs alone, involved in the phenomenon of charge transfer absorption in an iron-based coordination complex (Figure 4). The FT-IR analysis showed the characteristic signals of PLGA MVs at 1758, 1170, and 1091 cm^−1^, which, respectively, corresponded to the vibration in the C=O, C-O-C, and C-O bonds of PLGA (Figure 5). The new vibration signals of the CO triple bond (2009 cm^−1^) and Fe-C bond (640 and 477 cm^−1^) of IC were detected from IC-loaded PLGA MVs, indicating the presence of IC on the vesicles after the emulsion process. After characterization, all evidence indicated the presence of IC in the PLGA MVs, and these products showed excellent stability at low temperature (~4 °C), which is beneficial for long-term storage.

### 3.4. Evaluation of CO Release from IC-Loaded PLGA MVs

**Figure 6:**
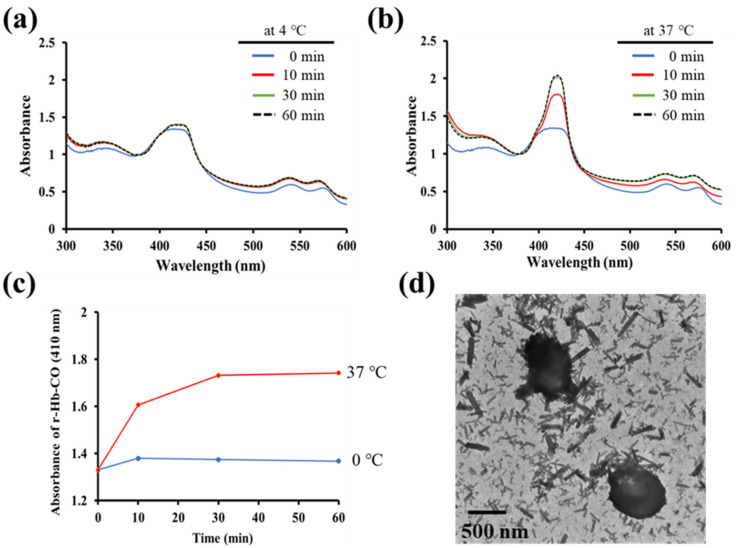
Evaluation of CO release from IC-loaded PLGA MVs. (**a**,**b**) The UV–vis spectra of reduced hemoglobin were measured to monitor the CO release from IC-loaded PLGA MVs at 4 and 37 °C for 0–60 min. (**c**) The dynamic CO release curves were obtained through the evaluation of the absorbance of r-Hb-CO at 410 nm. (**d**) A TEM image of the broken IC-loaded PLGA MVs.

A reduced hemoglobin (r-Hb) solution was prepared as an indicator to determine the amount of CO molecules and evaluate the CO-releasing capability of IC-loaded PLGA MVs [21]. The IC-loaded PLGA MVs were mixed with r-Hb at 4 and 37 °C for different incubation times (0, 10, 30, and 60 min). The thermally unstable IC molecules in the PLGA shell interior gradually decomposed into CO bubbles under warm conditions. Then, the stress increase caused by gas generation burst the vesicles, causing further gas release, and these free CO gases were captured by r-Hb to form the CO-conjugated r-Hb, termed r-Hb-CO. Afterwards, the mixture was centrifugated once, and UV–vis was used to further measure the supernatant of r-Hb-CO to determine the amounts of CO released from MVs. Figure 6a shows all the UV–vis spectra without obvious differences, demonstrating that the IC-loaded PLGA MVs do not release CO gas and are very stable at 4 °C. Interestingly, a significantly increased absorbance of r-Hb-CO at 410 nm was obtained after leaving the IC-loaded PLGA MVs in a warm environment for 10, 30, and 60 min (Figure 6b). CO generation gradually increased depending on the time of incubation at 37 °C, which supports the high applicability of IC-loaded PLGA MVs in spontaneously releasing CO under body temperature conditions (Figure 6c). The TEM image of the IC-loaded PLGA MVs incubated at 37 °C for 60 min showed the destruction of MVs, implying IC decomposition, CO bubble generation, and thus the destruction of the polymer shell (Figure 6d). We believe that the stability of the cargo is the critical factor affecting the behavior of CO release. Another relatively stable drug candidate, triiron dodecarbonyl (Fe_3_(CO)_12_), was loaded in the PLGA MVs following the same preparation method. No CO gas generation from carriers was measured by the same analysis system, even when maintained at 95 °C for 10 min (data are not shown here).

### 3.5. In Vitro Study for the Evaluation of CO-Induced Cytotoxicity by IC-Loaded PLGA MVs

**Figure 7:**
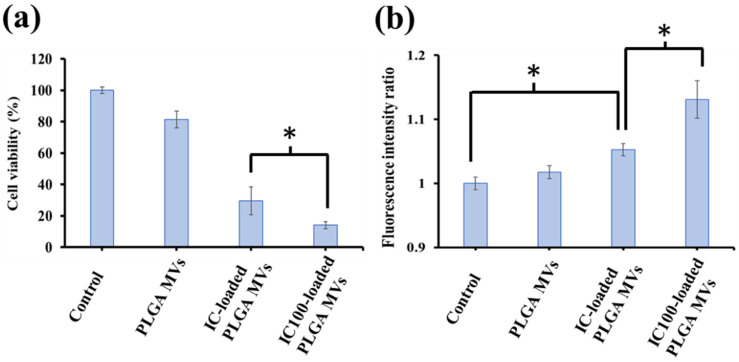
The in vitro study was used to evaluate the cytotoxicity of CO therapy. (**a**) The cell viability analysis of A549 cancer cells treated with medium alone (control), PLGA MVs, IC-loaded PLGA MVs, and IC100-loaded PLGA MVs. * *p* < 0.05, calculated and compared to IC-loaded and IC100-loaded PLGA MVs. (**b**) The intracellular ROS levels of A549 cancer cells treated with medium alone (control), PLGA MVs, IC-loaded PLGA MVs, and IC100-loaded PLGA MVs were determined by the fluorescence intensity of APF dye at 525 nm. * *p* < 0.05, calculated and compared to IC-loaded and IC100-loaded PLGA MVs as well as control and IC-loaded PLGA MVs.

For the next step, the applicability of IC-loaded PLGA MVs in drug delivery and CO therapy was checked. The human lung carcinoma/alveolar cell line (A549) was selected as the cell model in all in vitro studies. Through an MTT assay, cell viability was evaluated with and without IC-loaded PLGA MVs after 24-h incubations. The IC100-loaded and IC-loaded PLGA MVs showed an excellent ability to kill the cancer cells, resulting in cancer cell viabilities of 14 and 29%, respectively (Figure 7a). No significant cytotoxicity was observed after treatment with PLGA MVs alone, which reveals the high biocompatibility of this vesicular material. The cytotoxicity of the cells treated with IC-loaded PLGA MVs was due to the action of CO molecules, which not only generated high amounts of reactive oxygen species (ROS) but also induced mitochondrial exhaustion, which in turn induced the apoptosis of cancer cells [20]. APF dye was used as the ROS indicator to assess the ROS level inside the cells. Through analysis by a fluorescence reader, the A549 cells treated with IC-loaded and IC100-loaded PLGA MVs showed very strong fluorescence signals compared to IC-free groups, demonstrating the high ROS level inside the cells, which was activated by CO stimulation (Figure 7b). All in vitro outcomes demonstrated the highly efficient CO treatment of IC-loaded PLGA MVs and revealed that PLGA MVs are a feasible drug delivery system, especially as a carrier for delivering highly toxic and unstable coordination complexes.

### 3.6. The Future Development of Synthetic MVs

Even though the application of synthetic MVs is very restricted because they lack natural membrane proteins and biological cargos, they are still highly reliable as a vesicle-type DDS for delivering hydrophobic and unstable drugs. In addition, applying synthetic approaches would allow us to easily obtain MVs on a gram scale in a short time, which is impossible when harvesting MVs by biological methods. This advantage of the high yield is vital to provide an inexhaustible supply of synthetic MVs at a low cost for various clinical applications in the future. Notwithstanding that, these synthetic MVs still have much room for improvement, which would enable similar applications as biogenic MVs. To achieve this aim, many feasible strategies and concepts have been devised and reported in recent literature [17]. Inserting or attaching the functional proteins to the surface of synthetic MVs is a direct method to endow them with biological activity for additional purposes, such as immune activation, specific cell targeting, and intercellular communication [22,23]. As an example, our PLGA vesicles could form a corona through BSA adsorption onto the surface [24]. Using biological materials to replace the polymer to prepare MVs is a feasible way to reduce the differences between synthetic and biogenic MVs. Vesicles prepared with lipids and fatty acids showed excellent biocompatibility and could be a DDS [25,26]. An alternative strategy of integrating synthetic and biogenic MVs to form hybrid membrane MVs or natural membrane-coated MVs showed huge potential for future development [27,28,29]. On the other hand, the combination of synthetic MVs and functionalized nanoparticles with various features, such as hyperthermia or photothermal, photodynamic, photoacoustic effects, or magnetic targeting, among others, produced hybrid therapeutic platforms that could constitute a promising medical means to treat tumors in the future [30]. Accurate delivery of PLGA MVs to tumor tissue is a critical issue in actual future applications. Antibody-modified PLGA MVs and PLGA-magnetic nanobeads hybrid MVs are two feasible options to increase the homing efficiency of MVs into the tumor by specific bio-conjugation effect and remotely controllable magnetic targeting, respectively.

## 4. Conclusions

Our artificial MVs showed an excellent ability to carry unstable iron-based complexes and revealed significant cytotoxicity to lung cancer cells through spontaneous IC decomposition into CO at 37 °C and ensuing CO-related mitochondria exhaustion. This result indicates that PLGA MVs with cell membrane-mimic shells represent a suitable carrier for water-insoluble drug delivery. Notably, a synthetic approach for preparing MVs is more feasible for achieving a reasonable yield for clinical requirements than biogenic methods. However, this kind of synthetic PLGA MV does not have natural membrane proteins on the surface, resulting in the intrinsic disadvantage of lacking other biofunctions such as immune activation, cell targeting, microenvironment modulation, and cell-to-cell communication. This drawback significantly limits this polymeric material’s applicability in many potential fields. We believe that this limitation will probably be overcome through new technologies developed in the future. The modification of the surface of MVs by natural biological membranes or active proteins is a possible direction for future research in order to reduce the differences in the surface characteristics of vesicles created by synthetic and biogenic approaches.

## Data Availability

The data presented in this study are available on request from the corresponding author.

## References

[1] Raposo G., Stoorvogel W. (2013). Extracellular vesicles: Exosomes, microvesicles, and friends. J. Cell Biol..

[2] Hill E.H., Solomon P.S. (2020). Extracellular vesicles from the apoplastic fungal wheat pathogen *Zymoseptoria tritici*. Fungal Biol. Biotechnol..

[3] Niel G., D’Angelo G., Raposo G. (2018). Shedding light on the cell biology of extracellular vesicles. Nat. Rev. Mol. Cell Biol..

[4] Souza W., Barrias E.S. (2020). Membrane-bound extracellular vesicles secreted by parasitic protozoa: Cellular structures involved in the communication between cells. Parasitol. Res..

[5] Schwechheimer C., Kuehn M.J. (2015). Outer-membrane vesicles from Gram-negative bacteria: Biogenesis and functions. Nat. Rev. Microbiol..

[6] Paolicelli R.C., Bergamini G., Rajendran L. (2019). Cell-to-cell communication by extracellular vesicles: Focus on microglia. Neuroscience.

[7] Stotz H.U., Brotherton D., Inal J. (2021). Communication is key: Extracellular vesicles as mediators of infection and defence during host-microbe interactions in animals and plants. FEMS Microbiol. Rev..

[8] Chronopoulos A., Kalluri R. (2020). Emerging role of bacterial extracellular vesicles in cancer. Oncogene.

[9] Becker A., Thakur B.K., Weiss J.M., Kim H.S., Peinado H., Lyden D. (2016). Extracellular vesicles in cancer: Cell-to-cell mediators of metastasis. Cancer Cell.

[10] Herrmann I.K., Wood M.J.A., Fuhrmann G. (2021). Extracellular vesicles as a next-generation drug delivery platform. Nat. Nanotechnol..

[11] Kang M., Jordan V., Blenkiron C., Chamley L.W. (2021). Biodistribution of extracellular vesicles following administration into animals: A systematic review. J. Extracell. Vesicles.

[12] Park K.S., Svennerholm K., Crescitelli R., Lässer C., Gribonika I., Lötvall J. (2021). Synthetic bacterial vesicles combined with tumour extracellular vesicles as cancer immunotherapy. J. Extracell. Vesicles.

[13] Thonea M.N., Kwon Y.J. (2020). Extracellular blebs: Artificially-induced extracellular vesicles for facile production and clinical translation. Methods.

[14] Arab T., Mallick E.R., Huang Y., Dong L., Liao Z., Zhao Z., Gololobova O., Smith B., Haughey N.J., Pienta K.J. (2021). Characterization of extracellular vesicles and synthetic nanoparticles with four orthogonal single-particle analysis platforms. J. Extracell. Vesicles.

[15] Wang M., Zhou C., Chen J., Xiao Y., Du J. (2015). Multifunctional biocompatible and biodegradable folic acid conjugated poly(ε-caprolactone)−polypeptide copolymer vesicles with excellent antibacterial activities. Bioconjugate Chem..

[16] Kotla N.G., Chandrasekar B., Rooney P., Sivaraman G., Larrañaga A., Krishna K.V., Pandit A., Rochev Y. (2017). Biomimetic lipid-based nanosystems for enhanced dermal delivery of drugs and bioactive agents. ACS Biomater. Sci. Eng..

[17] Koog L., Gandek T.B., Nagelkerke A. (2021). Liposomes and extracellular vesicles as drug delivery systems: A comparison of composition, pharmacokinetics, and functionalization. Adv. Healthc. Mater..

[18] Motterlini R., Otterbein L.E. (2010). The Therapeutic potential of carbon monoxide. Nat. Rev. Drug Discov..

[19] Fujita K., Tanaka Y., Sho T., Ozeki S., Abe S., Hikage T., Kuchimaru T., Kizaka-Kondoh S., Ueno T. (2014). Intracellular CO release from composite of ferritin and ruthenium carbonyl complexes. J. Am. Chem. Soc..

[20] Wegiel B., Gallo D., Csizmadia E., Harris C., Belcher J., Vercellotti G.M., Penacho N., Seth P., Sukhatme V., Ahmed A. (2013). Carbon monoxide expedites metabolic exhaustion to inhibit tumor growth. Cancer Res..

[21] Wang S., Yuan F., Chen G., Tu K., Wang H., Wang L.Q. (2014). Dextran-based thermo-responsive hemoglobin–polymer conjugates with oxygen-carrying capacity. RSC Adv..

[22] Li S., Zhang Y., Wang J., Zhao Y., Ji T., Zhao X., Ding Y., Zhao X., Zhao R., Li F. (2017). Nanoparticle-mediated local depletion of tumour-associated platelets disrupts vascular barriers and augments drug accumulation in tumours. Nat. Biomed. Eng..

[23] Garni M., Thamboo S., Schoenenberger C.A., Palivan C.G. (2017). Biopores/membrane proteins in synthetic polymer membranes. Biochim. Biophys. Acta.

[24] Li W.P., Su C.H., Chang Y.C., Lin Y.J., Yeh C.S. (2016). Ultrasound-induced reactive oxygen species mediated therapy and imaging using a Fenton reaction activable polymersome. ACS Nano.

[25] Zhang Y., Wei J., Liu S., Wang J., Han X., Qin H., Lang J., Cheng K., Li Y., Qi Y. (2017). Inhibition of platelet function using liposomal nanoparticles blocks tumor metastasis. Theranostics.

[26] Douliez J.P., Gaillard C. (2014). Self-assembly of fatty acids: From foams to protocell vesicles. New J. Chem..

[27] Dehaini D., Wei X., Fang R.H., Masson S., Angsantikul P., Luk B.T., Zhang Y., Ying M., Jiang Y., Kroll A.V. (2017). Erythrocyte–platelet hybrid membrane coating for enhanced nanoparticle functionalization. Adv. Mater..

[28] Egloff-Juras C., Bezdetnaya L., Dolivet G., Lassalle H.P. (2019). NIR fluorescence-guided tumor surgery: New strategies for the use of indocyanine green. Int. J. Nanomed..

[29] Wang Y., Zhang K., Li T., Maruf A., Qin X., Luo L., Zhong Y., Qiu J., McGinty S., Pontrelli G. (2021). Macrophage membrane functionalized biomimetic nanoparticles for targeted anti-atherosclerosis applications. Theranostics.

[30] Li T., Qin X., Li Y., Shen X., Li S., Yang H., Wu C., Zheng C., Zhu J., You F. (2020). Cell membrane coated-biomimetic nanoplatforms toward cancer theranostics. Front. Bioeng. Biotechnol..

